# Molecular Dynamics Simulation of Oxidative Aging Effect on Diffusion Behaviors of Oxygen and Cyclohexane in NBR

**DOI:** 10.3390/polym14102060

**Published:** 2022-05-18

**Authors:** Jinsong Yang

**Affiliations:** School of Traffic and Transportation Engineering, Central South University, Changsha 410075, China; yangjs@csu.edu.cn; Tel.: +86-191-3070-8827

**Keywords:** molecular dynamics simulation, diffusion behavior, oxidative aging, aged model, J0101

## Abstract

The influences of thermal-oxidative aging on the diffusion behaviors of oxygen and cyclohexane in nitrile-butadiene rubber (NBR) at the micro-scale were investigated by molecular dynamics (MD) simulation. The two types of aged rubber models were established on the basis of rubber oxidative chains modified by the introduction of hydroxyl groups and carbonyl groups in rubber chains. The diffusion behaviors of oxygen and cyclohexane in NBR under different conditions were characterized by the fractional free volume (*FFV*), mean square displacement (*MSD*), diffusion coefficients, and diffusion trajectory. It turns out that the elevated temperature contributed to the increase in the free volume and diffusion range of oxygen and cyclohexane, while the compressive stress showed the reverse influence. Additionally, the introduction of oxidative polar functional groups (hydroxyl groups and carbonyl groups) in rubber chains lowered the flexibility of the rubber chains and promoted the formation of strong polar interaction, which further inhibits the diffusion of oxygen and cyclohexane.

## 1. Introduction

Due to the excellent properties of oil resistance, anti-heat aging, and high permanent set resistance, the nitrile-butadiene rubber (NBR) products such as seals, gaskets, and hoses are widely used in automotive and aeronautic industries to inhibit the leakage of liquids and gases [1,2,3,4]. Under the actual service conditions, nitrile rubber products are usual subjected to the coupled effects of mechanical stress, oxygen, elevated temperature, radiation, and liquid mediums, resulting in the degradation of the microstructure and mechanical performances of the rubber products over a long-term [5,6,7]. The on-going deterioration of rubber products may lead to the sealing failure of functional parts, for example, the leakage of gases and liquids [8,9,10].

The degradation behavior and mechanism of nitrile rubber materials or products under environmental conditions such as temperature, mechanical stress, radiation, atmosphere, and chemical medium have been investigated for decades [11,12,13,14,15]. The typical degradation mechanism of NBR exposed to air is thermo-oxidative aging related to the chemical reaction between the polymer matrix and oxygen. Moreover, the typical reaction process consists of crosslinking, chain scission, and the oxidation of molecular chains, accompanied by the formation of oxidation products such as hydroxyl groups, carbonyl groups, and hyperoxide [16,17,18]. If the rubber products are simultaneously exposed to other gases or immersed in liquid mediums, more complicated chemical reactions will occur due to the interactions between polymer chains and gas or liquid molecules [19,20]. Additionally, the liquid medium can also swell the rubber network structure and extract the additives of rubber, leading to the further degradation of rubber materials [21,22]. Obviously, the gas or liquid molecules have a significant effect on the physical and chemical changes during the oxidative aging process [23,24,25]. Meanwhile, the formation of oxygen-containing groups can change the physicochemical properties of the rubber matrix, which may play a positive or negative role in the diffusion mechanism of gases and liquid medium into the rubber matrix, further accelerating or restraining the oxidative reaction rate [20,26,27].

Many researchers have focused on the diffusion behavior and mechanism of gas and liquid molecules in a rubber matrix through experiments and simulations [28,29,30,31,32]. Li et al. [33] studied the diffusion behavior of oil in natural rubber at different temperatures and pressures by MD simulation. They found that the diffusion coefficients of cyclohexane molecules increased with temperature, while the pressure slightly affected the cyclohexane molecules. Kucukpinar et al. [34] investigated the gas molecule (H_2_, O_2_, CO_2_, CH_4_) diffusion and solubility in three kinds of polymer materials through molecular simulations. They concluded that the diffusion coefficients of the gases decreased in the following order: SBR (poly (styrene-stat-butadiene)rubber) > PS (atactic polystyrene) > SMA (poly(styrene-alt-maleic anhydride) copolymer), while the solubility coefficients showed the opposite order. Zhi et al. [35] studied the oxygen diffusion behaviors in natural rubber by using molecular simulation. It can be found that the heterogeneous degradation caused by diffusion-limited oxidation can result in a complex stress distribution in natural rubber. Hence, during the oxidative aging process, a fundamental understanding of the diffusion behavior and mechanism of gas molecules and liquid molecules in the aged rubber is still incomplete and need to be further investigated by considering the rubber oxidation aging effect.

The aging behavior of rubber products exposed to air and hydraulic oil at different temperatures was investigated using the accelerated aging test in our previous studies [36,37]. The thermal oxidative aging behavior and mechanism were deeply understood such as the chemical reactions (crosslinking and chain scission) and the formation of oxidation products. In this study, the effects of the oxidation products (hydroxyl groups and carbonyl groups) on the diffusion behaviors of the oxygen and hydraulic oil in the rubber matrix under different temperatures and compression sets were investigated by the MD simulations. The different oxidized chains were established by adopting hydroxyl groups and carbonyl groups to modify the molecular chains, respectively. Then, the aged rubber models were established on the basis of the rubber oxidized chains and small molecules. After that, the molecular dynamics simulations were conducted. By analyzing the fractional free volume, mean square displacement, self-diffusion coefficients, diffusion trajectory, the influence of the oxidative aging of the rubber chains on the diffusion behaviors of the oxygen and hydraulic oil in the rubber matrix under different conditions at the molecular scale was investigated.

## 2. Simulation Models and Methods

The MD simulations were conducted using the Forcite and Amorphous cell modules of the Materials Studio 8.0. The COMPASS (Condensed-Phase Optimized Molecule Potentials for Atomistic Simulation Studies) force field was used for all the theoretical calculations including the optimization and prediction of the structural, conformational, and thermophysical condensed phase properties of molecules such as small inorganic molecules and polymers.

### 2.1. Construction of the Unaged and Aged NBR Models

To study the influence of oxidative aging on the diffusion behaviors of oxygen and hydraulic oil in the aged nitrile rubber at the molecular level, different molecular models were built in this study, respectively.

**Figure 1:**
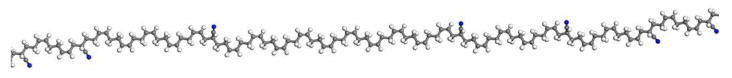
The unaged nitrile rubber molecular chain.

**Figure 2:**
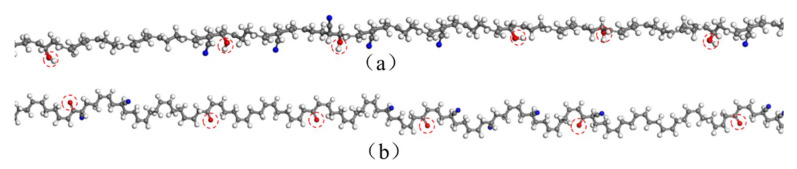
The aged nitrile rubber molecular chain: (**a**) modified by the hydroxyl groups; (**b**) modified by the carbonyl groups.

According to the oxidative aging mechanism discovered in previous studies [17,38,39], the aging mechanism of NBR was mainly constituted of thermal oxidative aging at elevated temperature, accompanying the formation of oxygen-containing functional groups. The typical oxygen-containing functional groups were the hydroxyl groups and carbonyl groups. The formation of the oxidation products during aging directly changed the physicochemical properties of the rubber molecular chains, which further led to the changes in the structure and properties of the rubber materials. Thus, to estimate the oxidative aging effect, the hydroxyl groups and carbonyl groups were considered and used to modify the possible oxidizable locations in the molecular chains, representing the oxidative aging of the amolecular chains. The unaged rubber molecular chain containing 35% acrylonitrile was constituted of 50 atomistic repeating units of butadiene and acrylonitrile, as shown in Figure 1. Then, the unaged molecular chains were modified by the introduction of the hydroxyl groups and carbonyl groups, respectively, as shown in Figure 2. It should be noted that the oxidative aging mechanism of NBR is still under exploration and the assumptions of the molecular structures after oxidation made in this study still need further investigation such as crosslinking and chain scission. In addition, fillers are often added and dispersed in the rubber matrix to improve the physical and mechanical properties of the rubber products, which can significantly affect the diffusion of oxygen and hydraulic oil. However, the thermal-oxidative aging may induce the volatilization, migration, and aggregation and the changes in the surface properties (e.g., click modified nano silica) of the fillers. The coupling effect of the oxidation of the molecular chains and the changes in the fillers can result in the poor compatibility between the rubber matrix and fillers. Thus, the effect of the changes in the fillers induced by thermal-oxidative aging on the diffusion behaviors of oxygen and hydraulic oil should be studied in further work.

The unaged and aged NBR models were established, respectively, based on the unaged and aged rubber chains. Moreover, the unaged NBR model was defined as “NBR”. The aged NBR model modified by hydroxyl groups was defined as “OH-NBR”. The aged NBR model modified by carbonyl groups was defined as “CO-NBR”. Meanwhile, to consider the effects of compression deformation on the diffusion behaviors of the oxygen and hydraulic oil in unaged and aged nitrile rubber, the unaged and aged NBR models were compressed to 70% of their original height based on the actual service condition [40]. The compression deformation was applied along the Z-direction. Additionally, the X-direction and Y-direction were constrained to change the shape and size of the unaged and aged NBR models.

### 2.2. MD Simulation Strategies

To study the diffusion behaviors of the oxygen and hydraulic oil in the unaged and aged NBR, a series of the periodic cells containing five rubber chains, eight oxygen molecules, and four cyclohexane molecules were established, respectively. The cyclohexane was used to represent the typical structure of the hydraulic oil. For the unaged and aged NBR models, the 1,000,000-step minimization was conducted by using the Smart Minimizer algorithm. Then, the cells were annealed at 0.1 MPa from 600 K to 300 K for 200 ps. After that, 200 ps of the NPT (constant number of particles, pressure, and temperature) simulation was performed at 300 K and 2 GPa, 1 GPa, 0.5 GPa, respectively, to further relax the network structure. Consequently, 500 ps of the NVT (constant number of particles, volume, and temperature) simulation was carried out at 300 K. Then, 500 ps of the NPT simulation was performed at 300 K and 101 KPa to gain a stable structure. The Andersen thermostat [41] and Berendsen barostat [42] were used to maintain the temperature and pressure, respectively. Finally, the 500 ps NVT product run was conducted at 101 kPa and 298.15 K (25 °C), 343.15 K (70 °C), 363.15 K (90 °C), and 383.15 K (110 °C), respectively, to achieve the final stable structure. These temperatures were selected according to the accelerated test plans in our previous study [37]. Then, the equilibrated cells can be applied to analyze the *FFV*, *MSD*, self-diffusion coefficients, and diffusion trajectory to further investigate the effect of oxidative aging on the diffusion behaviors of the oxygen molecules and cyclohexane molecules in the unaged and aged nitrile rubber.

## 3. Results and Discussion

### 3.1. Fractional Free Volume (FFV)

*FFV* is widely applied to estimate the diffusion behavior of the gas and liquid molecules in materials, representing the fraction of the volume not occupied by the polymer. The *FFV* can be obtained by the following equation:

(1) $$FFV=1-\frac{V_{0}}{V_{S}}$$

where the occupied volume $V_{0}$ = 1.3 $V_{W}$, and $V_{W}$ and $V_{S}$ correspond to the van der Waals’s volume and the specific volume, respectively.

**Figure 3:**
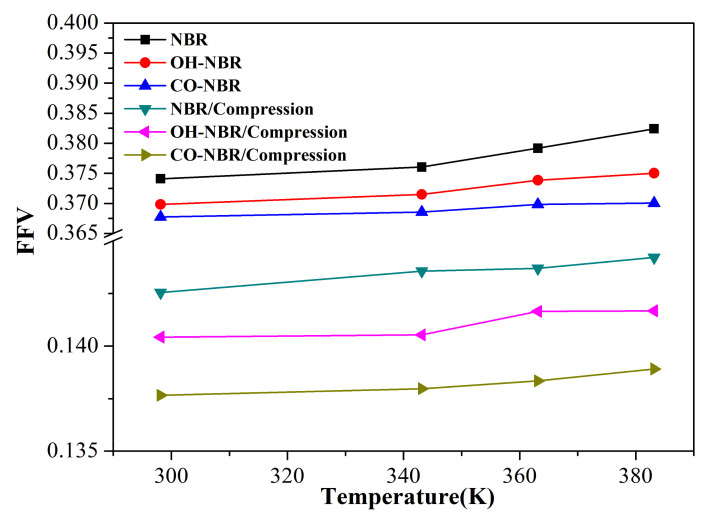
The *FFV* of the unaged and aged NBR models under the different conditions.

Figure 3 shows the *FFV* of the unaged and aged NBR models under the different conditions. The *FFV* all displayed an increase with an increase in the temperature. Additionally, the *FFV* of the uncompressed rubber model was higher than that of the compressed rubber model. The order of the *FFV* for the unaged and aged NBR models was *FFV*_NBR_ > *FFV*_OH-NBR_ > *FFV*_CO-NBR_. The free volume was mainly attributed to the thermal movement of the molecular chains. The higher temperature could intensify the mobility of the molecular chains and chain segments, resulting in the increase in the *FFV*. Additionally, under the compression state, the mobility of the molecular chains was weakened, and molecular packing occurred, which both resulted in the decrease in free volume. Moreover, there were strong polar interactions between the oxidized molecular chains due to the introduction of the hydroxyl groups and carbonyl groups. In particular, the rubber chains modified by the carbonyl groups, the stiffness of the backbone of the long rubber chains shows a significant increase lowering the mobility of rubber chains, which resulted in a gradual decrease in the *FFV*. These phenomena demonstrate that the compressive deformation and oxidation functional groups both display a negative effect on the free volume.

### 3.2. Mean Square Displacement (MSD) and Self-Diffusion Coefficients of Oxygen

**Figure 4:**
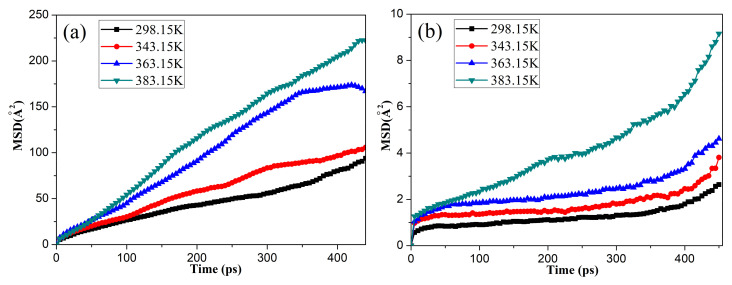
Changes in the mean square displacement (*MSD*) of the oxygen molecules in the unaged NBR under the free (**a**) and compression (**b**) states.

To investigate the influence of temperature on the diffusion behaviors of the oxygen molecules in the unaged NBR, the *MSDs* of the oxygen molecules in rubber under the free and compression state were calculated over a series of temperatures, as shown in Figure 4. The results show that the *MSD* of the oxygen molecules increased with the simulation time and temperature. Moreover, the *MSD* of the oxygen molecules in the NBR under the free state was significantly greater than that of the oxygen molecules under the compression state at the same temperature. All of these phenomena indicate that the higher temperature could accelerate the diffusion of oxygen molecules in the NBR, and the compressive deformation seriously restricted the diffusion of oxygen molecules in the matrix.

To further analyze the diffusion of the oxygen molecules in the matrix, the diffusion coefficient (D) of the oxygen molecules in the rubber was calculated by the Einstein equation [33]:

(2) $$D=\frac{1}{6N}\underset{t\to \infty }{\lim}\frac{d}{dt}\sum_{i=1}^{N}\langle {\left|r_{i}\left(t+t_{0}\right)-r_{i}\left(t_{0}\right)\right|}^{2}\rangle$$

where *N* is the number of molecules; *D* is the diffusion coefficients of the rubber molecular chains; $r_{i}\left(t_{0}\right)$ is the displacement at *t*_0_; $r_{i}\left(t+t_{0}\right)$ is the displacement at *t*_0_+*t*; and

(3) $$MSD\left(t\right)=\frac{1}{N}\sum_{i=1}^{N}\langle {\left|r_{i}\left(t+t_{0}\right)-r_{i}\left(t_{0}\right)\right|}^{2}\rangle$$

**Figure 5:**
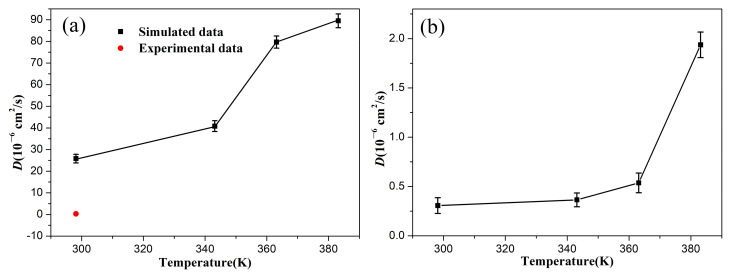
The diffusion coefficients of the oxygen in the uncompressed (**a**) and compressed (**b**) rubber as a function of temperature.

Figure 5 shows the diffusion coefficients of the oxygen in the uncompressed and compressed rubber at different temperatures. The diffusion coefficients of the oxygen molecules in the rubber at 298.15 K was evaluated as 25.83 × 10^−6^ cm^2^/s, which was larger than the ex-experimental results (0.28 × 10^−6^ cm^2^/s) [43]. The main reason is that no fillers were added into the rubber matrix of the constructed NBR model during the MD simulations. The results indicate that the diffusion coefficients of the oxygen molecules in the rubber increased with an increase in temperature, while the increase in the compression deformation led to the rapid decrease in the diffusion coefficient. This is mainly because the higher temperature improved the mobility of the rubber chains, which gave the oxygen molecules a higher energy to conquer the activation energy required to enter the new free volume voids [44]. However, the compressive strain seriously restricted the mobility of the molecular chains and segments, resulting in less free volume between the chains. These behaviors demonstrate that the higher temperature promoted oxygen diffusion during the aging process and accelerated the degradation of the rubber network structure through oxidative reactions, while the compression deformation showed the opposite effect, which well explained why the compression set and mechanical properties of the rubber seals showed more serious degradation at higher temperatures, but presented relatively slight degradation in our previous experimental results [37].

**Figure 6:**
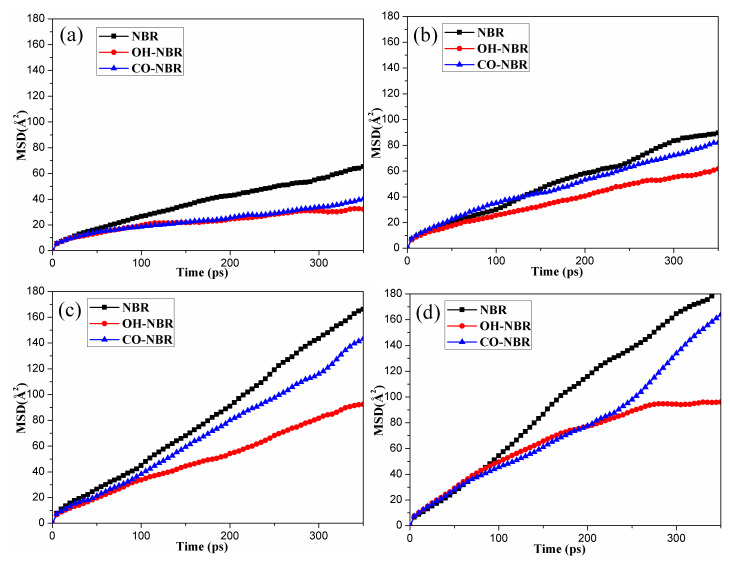
The *MSD* of the oxygen molecules with simulation time in the unaged and aged NBR models: (**a**) 298.15 K; (**b**) 343.15 K; (**c**) 363.15 K; (**d**) 383.15 K.

To further study the influence of oxidative aging (the formation of hydroxyl and carbonyl groups during oxidative aging process) on the diffusion behaviors of the oxygen molecules in rubber, the changes in the *MSD* with the simulation time in the different unaged NBR models were calculated, as shown in Figure 6. It was found that the *MSDs* of the oxygen molecules increased rapidly with the simulation time and temperature in the OH-NBR and CO-NBR models. Additionally, it could be clearly observed that for most cases at the same temperature, the order of the *MSDs* of the oxygen molecules in different oxidation aging models was *MSD*_NBR_ > *MSD*_CO-NBR_ > *MSD*_OH-NBR_.

**Figure 7:**
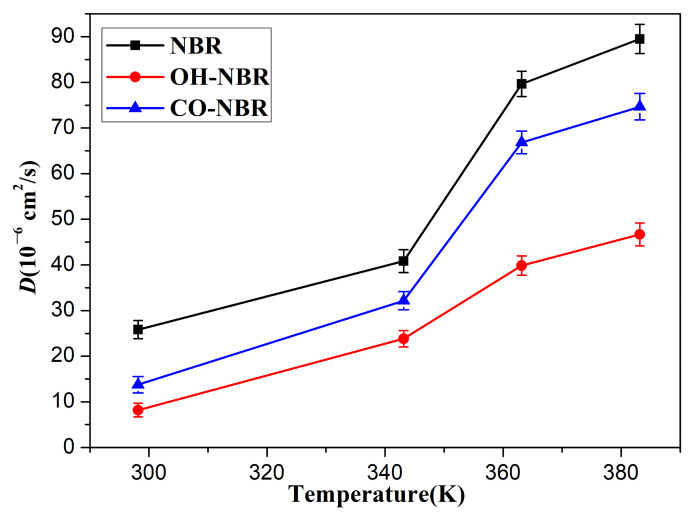
The diffusion coefficients of the oxygen molecules in the unaged and aged NBR models at different temperatures.

Figure 7 shows the diffusion coefficients of the oxygen molecules in the unaged and aged NBR models under different temperatures. It can be found that the diffusion coefficient of the oxygen molecules presented a gradual increase with an increase in temperature, and the order of the diffusion coefficients of the oxygen molecules in the unaged and aged NBR models was D_NBR_ > D_CO-NBR_ > D_OH-NBR_. These phenomena imply that the formation of polar oxidation functional groups in rubber chains limited the diffusion of oxygen molecules during the oxidative aging process. This can be explained by the introduction of hydroxyl groups and carbonyl groups in the rubber chains, raising the rigidity of the molecular chains and forming polar interactions and hydrogen bonds between the polar groups in oxidized chains, which lower the mobility of rubber molecular chains [38]. However, the diffusion of the oxygen molecules in rubber strongly depend on the mobility of molecular chains. Meanwhile, it was obviously found that the effect of hydroxyl groups on the diffusion behavior of the oxygen molecules was greater than that of the carbonyl groups due to the formation of hydrogen bonds between the oxidized chains modified by the hydroxyl groups.

### 3.3. Trajectory Analysis of Oxygen

**Figure 8:**
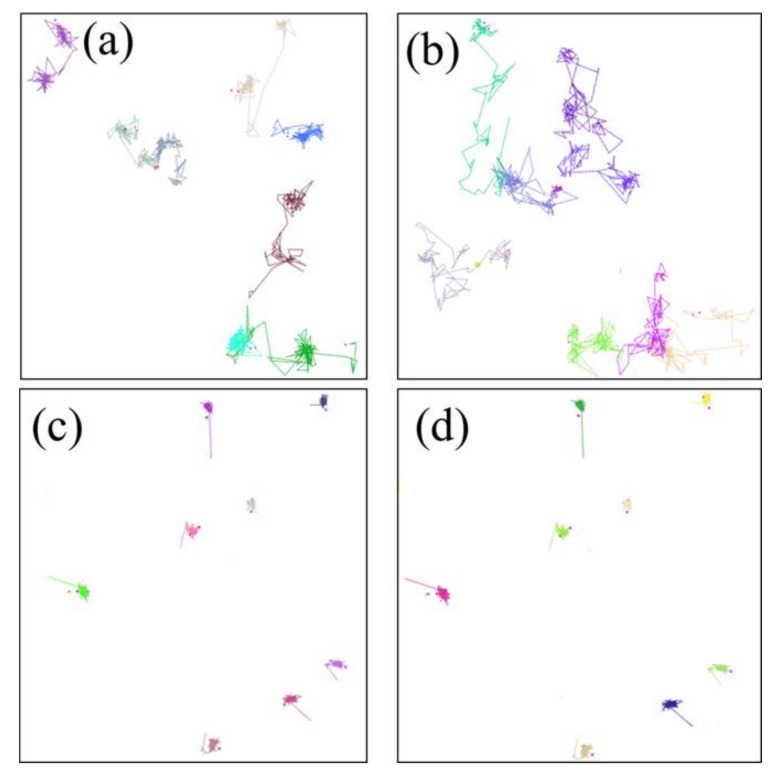
The motion trajectories of the oxygen molecules in the NBR: (**a**) 298.15 K/under the free state; (**b**) 383.15 K/under the free state; (**c**) 298.15 K/under the compression state; (**d**) 383.15 K/under the compression state. (Each color plot represents a motion trajectory of oxygen molecule).

Figure 8 shows the motion trajectories of the oxygen molecules in the uncompressed and compressed NBR at 298.15 K and 383.15 K. The color lines display the total diffusion process of the oxygen molecules in the rubber matrix. It can be observed that two types of basic diffusion modes of oxygen molecules occurred in NBR: (1) the diffusion route of the oxygen molecules presented frequent vibration in some local places; (2) oxygen molecules jumped from one local place to another. These results indicate that the diffusion of the oxygen molecules in the rubber was probably dominated by a mechanism of mixed diffusion modes. Moreover, at higher temperatures, the diffusion of the oxygen molecules displayed more jump events and a larger jump distance between the frequently probed places, implying that the second diffusion mode dominated. However, under the compression state, the diffusion mechanism of the oxygen molecules followed the first diffusion mode. Meanwhile, no obvious improvement occurred at higher temperatures. These phenomena indicate that the compression deformation greatly limited the diffusion of the oxygen molecules and inhibited the occurrence of the oxidation reaction.

**Figure 9:**
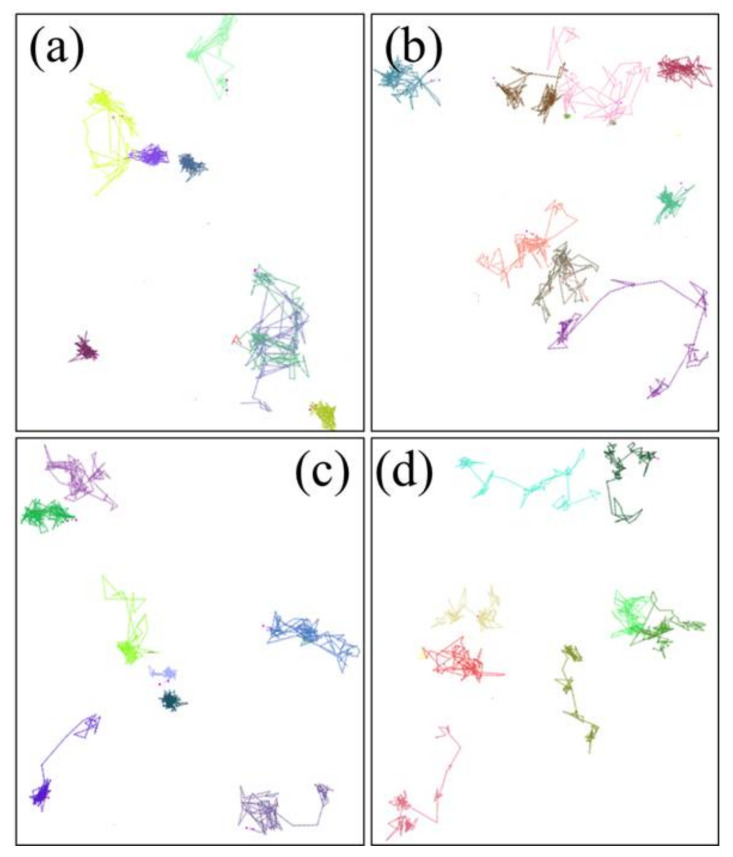
The motion trajectories of the oxygen molecules in the aged NBR models: (**a**) 298.15 K/OH-NBR; (**b**) 383.15 K/OH-NBR; (**c**) 298.15 K/CO-NBR; (**d**) 383.15 K/CO-NBR.

Figure 9 shows the motion trajectories of the oxygen molecules in the aged NBR models at different temperatures. Compared to the trajectories of the oxygen molecules in the unaged NBR model, it was found that the diffusion of the oxygen molecules in the aged NBR models at 298.15 K displayed more frequent vibrations at local places (namely the first diffusion mode), and could be more obviously observed in the OH-NBR model. Meanwhile, with the temperature increase, the first diffusion mode still occurred, but most oxygen molecules tended to conduct the second diffusion mode. Moreover, the order of the diffusion distance of the oxygen molecules in the unaged and aged NBR model was: D_NBR_ > D_CO-NBR_ > D_OH-NBR_. This evidence also indicates that the oxidized molecular chains restricted the diffusion of the oxygen molecules due to the increase in the inflexibility of the rubber chains and polar interaction between the molecular chains. These phenomena imply that the formation of the oxidation functional groups in the molecular chains can further inhibit the oxidation reaction rate by limiting the diffusion of oxygen molecules.

### 3.4. Mean Square Displacement (MSD) and Self-Diffusion Coefficients of Cyclohexane

**Figure 10:**
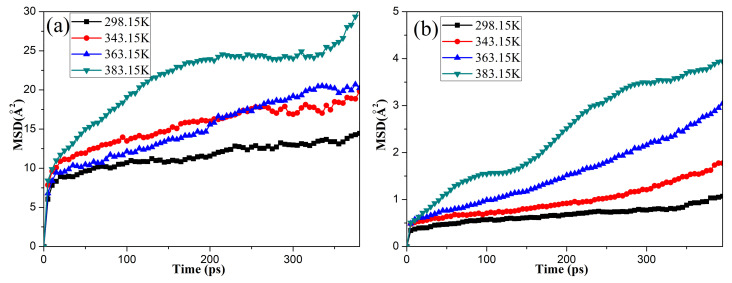
Changes in the mean square displacement (*MSD*) of the cyclohexane molecules in the unaged NBR under the free (**a**) and compression (**b**) states.

Figure 10 shows the *MSDs* of the cyclohexane molecules in the uncompressed and compressed NBR at different temperatures. It can be seen that the *MSD* of the cyclohexane molecules increased with an increase in the simulated time and temperature. Meanwhile, at the same temperature, the *MSD* of the cyclohexane molecules in the uncompressed NBR displayed a larger value compared with that of the oxygen molecules under the compression state. This evidence indicates that the high temperature could improve the mobility of the cyclohexane molecules, while the compression deformation greatly inhibited the diffusion of the cyclohexane molecules.

**Figure 11:**
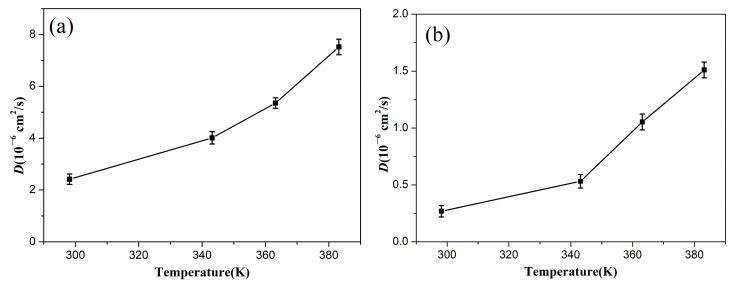
The diffusion coefficients of the cyclohexane molecules in the uncompressed (**a**) and compressed (**b**) rubber at different temperatures.

Figure 11 presents the diffusion coefficients of the cyclohexane molecules in the uncompressed and compressed NBR at different temperatures under the free and compression states, which were calculated by the Einstein equation. It was found that the diffusion coefficient of the oxygen molecules in the rubber increased with an increase in temperature while the diffusion coefficient decreased, which was caused by compressive stress. These phenomena demonstrate that the compression deformation greatly limited the diffusion of cyclohexane molecules in the rubber, and further inhibited the extraction of additives and swelling of the network structure in rubber, displaying the good agreement with our previous study [37]. By comparing the diffusion coefficients between the oxygen and cyclohexane, it was found that the *MSDs* and diffusion coefficients of the oxygen molecules in rubber were obviously higher than that of cyclohexane under the same conditions. This may be due to the larger volume of the cyclohexane molecule, while the rubber model has more and smaller free volume voids contributing to the diffusion of oxygen molecules, and the larger free volume voids are few in number, leading to the poor connectivity between voids. Therefore, when the higher temperature contributes to sufficient larger free volume voids to connect each other easily to form large nonselective voids, the cyclohexane molecules can enhance the probability of diffusion through the “channels” between these free volume voids [45]. Additionally, based on the analysis, it can be concluded that the *MSDs* and diffusion coefficients of the cyclohexane molecules at different temperatures showed little difference, which can well explain the phenomenon where the mass loss of the rubber seals immersed in the hydraulic oil displayed a similar trend of changes at different temperatures in our previous study [36].

**Figure 12:**
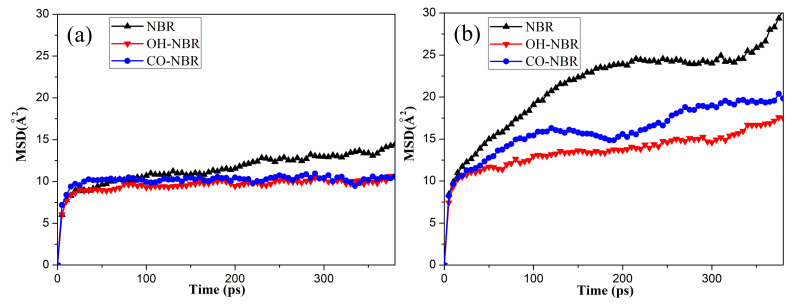
The *MSD* of the cyclohexane molecules with the simulation time in the unaged and aged NBR models: (**a**) 298.15 K; (**b**) 383.15 K.

**Figure 13:**
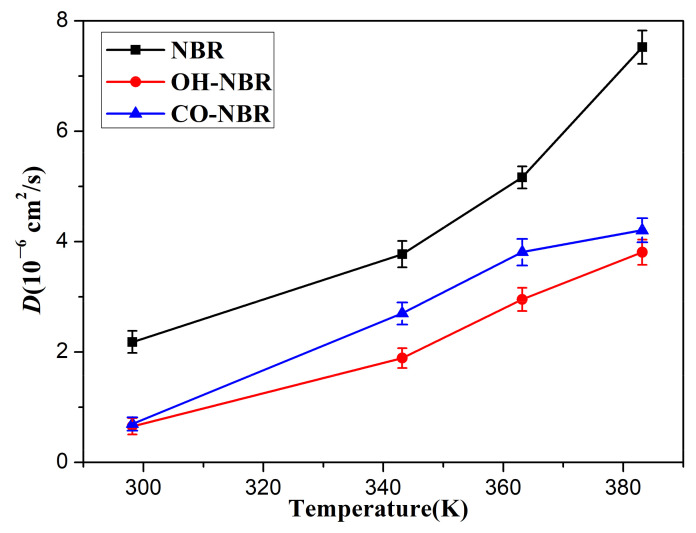
The diffusion coefficients of the cyclohexane molecules in the unaged and aged NBR models at different temperatures.

Figure 12 and Figure 13 show the *MSD* and diffusion coefficients of the cyclohexane molecules in the unaged and aged NBR models at different temperatures, respectively. It can be observed that the *MSD* and diffusion coefficient of the cyclohexane molecules both increased with temperature. Additionally, the order of *MSDs* and the diffusion coefficients of the cyclohexane molecules in the unaged and aged NBR models was: NBR > CO-NBR > OH-NBR. These phenomena imply that the oxidative aging of the rubber chains modified by the hydroxyl groups and carbonyl groups increased the inflexibility, resulting in the decrease in the mobility and contributed to form stronger polar interactions. These effects decreased the free volume fraction in rubber models, which resulted in the lower probability for forming the large free volume voids. Meanwhile, due to the large volume and low mobility of cyclohexane, strong polar interactions may form between polar groups and cyclohexane, leading to a further decrease in the mobility of cyclohexane. These may be the main reasons why the *MSDs* and diffusion coefficients of cyclohexane were significantly lower than that of oxygen under the same conditions.

### 3.5. Trajectory Analysis of Cyclohexane

**Figure 14:**
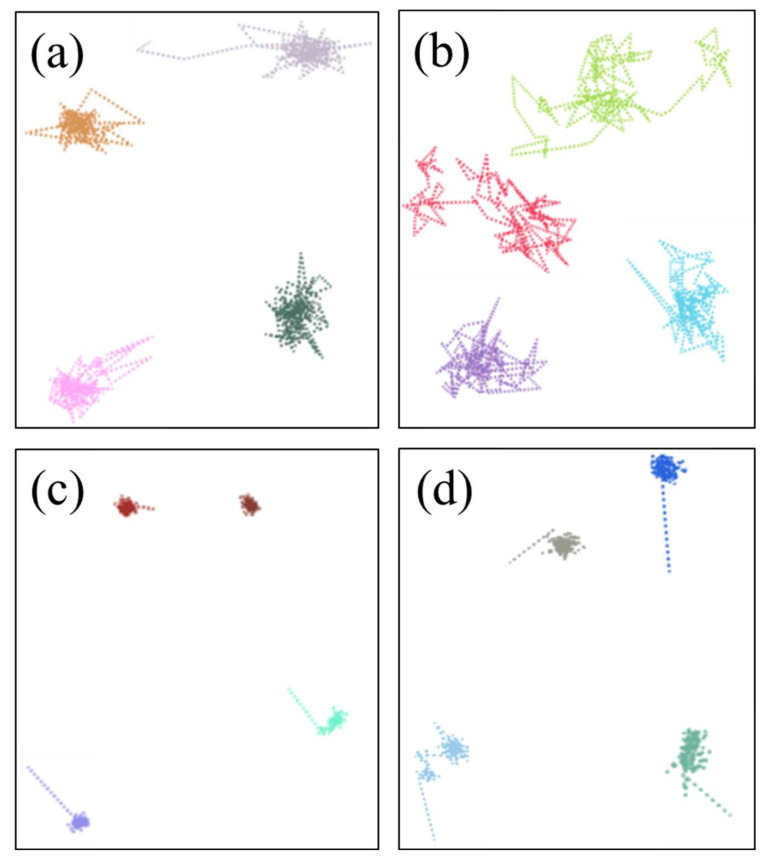
The motion trajectories of the cyclohexane molecules in the NBR: (**a**) 298.15 K/under the free state; (**b**) 383.15 K/under the free state; (**c**) 298.15 K/under the compression state; (**d**) 383.15 K/under the compression state. (Each color plot represents a motion trajectory of cyclohexane molecule).

Figure 14 shows that the motion trajectories of cyclohexane molecules at 298.15 K and 383.15 K in the uncompressed and compressed NBR system model. The results show that the diffusion modes of cyclohexane molecules also displayed two types of basic diffusion modes like the diffusion modes of oxygen molecules. However, at lower temperature and under the compression state, the frequently vibration of cyclohexane molecules in some local places dominated, while cyclohexane molecules showed more jump events at higher temperature. These phenomena indicate that the high temperature enhanced the diffusion distance of the cyclohexane molecules, but displayed a lesser effect under the compression state.

**Figure 15:**
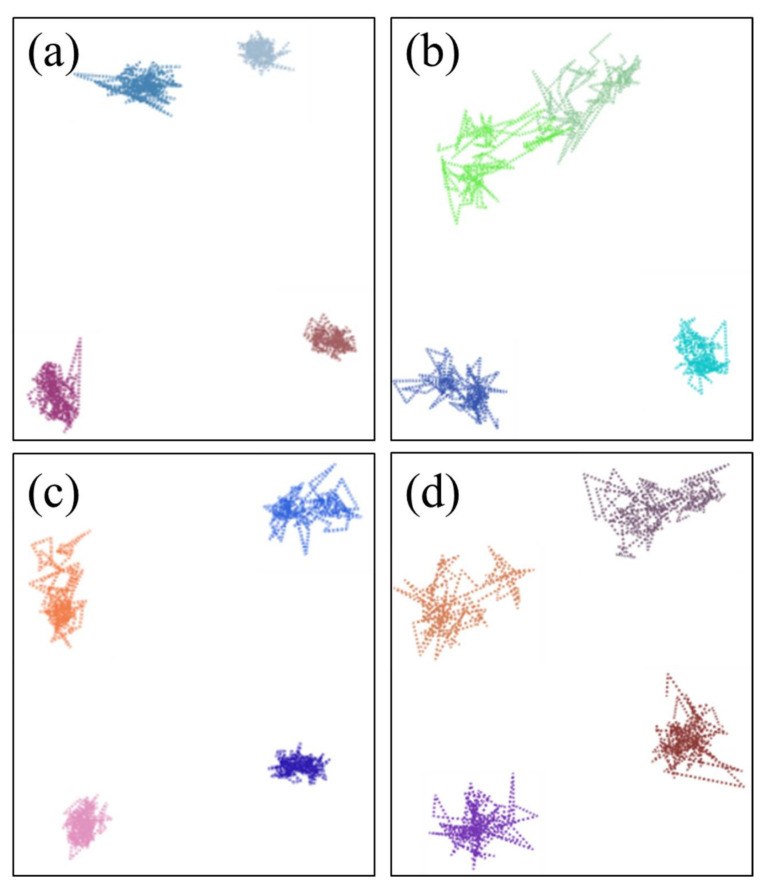
The motion trajectories of the cyclohexane molecules in the aged NBR models: (**a**) 298.15 K/OH-NBR; (**b**) 383.15 K/OH-NBR; (**c**) 298.15 K/CO-NBR; (**d**) 383.15 K/CO-NBR.

The motion trajectories of the cyclohexane molecules in the aged NBR models at different temperatures are presented in Figure 15. It can be seen that the diffusion modes of the cyclohexane molecules in the aged NBR models were similar to that of the cyclohexane molecules in the unaged NBR models at low and high temperatures. However, the cyclohexane molecules in the unaged NBR models still displayed a larger jump distance. An explanation for the phenomenon is that the formation of the oxidation functional groups during the oxidative aging process greatly limited the diffusion of the cyclohexane molecules due to the lower mobility of the molecular chains and stronger interaction between the oxidative molecular chains.

## 4. Conclusions

The diffusion behaviors of oxygen and cyclohexane in the unaged and aged nitrile rubber was investigated through MD simulation. The following conclusions can be concluded from the analysis:

1. The *FFV* results indicate that the *FFV* increased with temperature, while it decreased accompanying the introduction of the compressive strain and the oxidation functional groups in the rubber chains.
2. The diffusion behaviors of the oxygen and cyclohexane demonstrate that the high temperature significantly promoted the diffusion of two molecules in the rubber, while the compression deformation seriously restricted the diffusion behavior. Additionally, the larger volume of the cyclohexane molecules displayed the lower diffusion ability compared with the oxygen molecules. The diffusion phenomena of oxygen and cyclohexane in the unaged and aged NBR indicate that the formation of polar oxidation groups in the rubber molecular chains inhibited the diffusion of two molecules, and the hydroxyl groups showed a negative effect. These phenomena indicate that the high temperature contributed to the destruction of the rubber network structure caused by the oxygen molecules, while the compression deformation and the formation of the oxidation functional groups inhibited the damage.

## Data Availability

Data are contained within the article.
